# Supplementary figures and images for: The burning island: Spatiotemporal patterns of fire occurrence in Madagascar
Source: PLoS One. 2022 Mar 31;17(3):e0263313. doi: 10.1371/journal.pone.0263313 (PMC8970516; doi:10.1371/journal.pone.0263313)

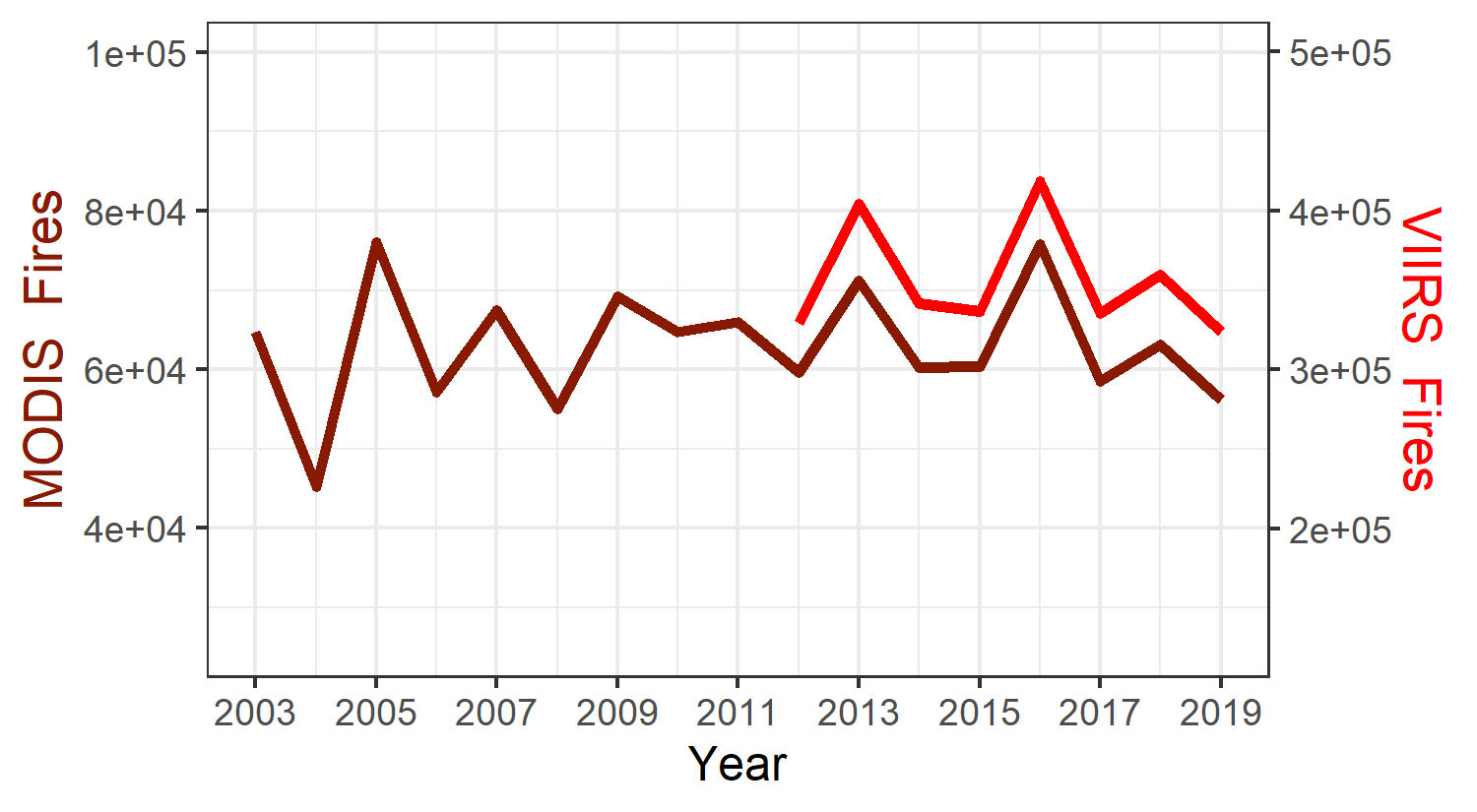

Supplement: S1 Fig — Total number of fires detected by MODIS (left axis) from 2003–2019 and VIIRS (right axis) from 2012–2019. VIIRS detected an average of 5.6 times more fires than MODIS. (TIF) [file pone.0263313.s001.tif]
