# Supplementary material for: The burning island: Spatiotemporal patterns of fire occurrence in Madagascar
Source: PLoS One. 2022 Mar 31;17(3):e0263313. doi: 10.1371/journal.pone.0263313 (PMC8970516; doi:10.1371/journal.pone.0263313)
Supplement: S1 File — (PDF) [file pone.0263313.s010.pdf]


From: Graves, Kate <Kate.Graves@wwf.org>
Sent: December 7, 2021 11:45 AM
To: Tristan Frappier-Brinton <tristan.frappierbrinton@duke.edu>
Cc: Buchner, Daniel <Daniel.Buchner@wwfus.org>; Thau, David <dave.thau@wwf.org>
Subject: RE: [EXTERNAL]Permission to use WWF Ecoregions data

Hello Tristan,

Thank you for doing your due diligence and reaching out to obtain permission for use of this data. On behalf of WWF, I am able to approve your request to use the Terrestrial Ecoregions of the World ecoregion data under the CC By 4.0 terms.

You may use this email to verify permission has been granted.

Best of luck with publishing your manuscript and your academic pursuits.

Cheers,
Kate

Kate Graves | Director | Global Science Communications and Fuller Program
skype k8graves | @KateGraves
She/her pronouns

From: Tristan Frappier-Brinton <tristan.frappierbrinton@duke.edu>
Date: Tuesday, November 23, 2021 at 11:25 AM
To: Thau, David <dave.thau@wwf.org>
Subject: [EXTERNAL]Permission to use WWF Ecoregions data

CAUTION: This email originated from outside of the organization. Do not click links or open attachments unless you recognize the sender and know the content is safe.

Hello,

I am a PhD student researcher at Duke University, and I am currently working to publish a manuscript on the patterns of fire use in Madagascar. I was hoping to publish this work in PLOS ONE so that it can be made accessible to local Malagasy researchers and conservation workers who may not have institutional access, but I learned that PLOS ONE publishes under a CC BY 4.0 license, whereas the WWF Terrestrial Ecoregions of the World data that I used in my analysis is only available under CC BY 3.0. So, I was hoping that you could help me obtain permission to use the WWF Ecoregion data in the publication I am working on, which would require approval from a WWF representative.

The general outlines of the ecoregions used by the WWF have been used in publications already published in PLOS ONE (e.g. https://journals.plos.org/plosone/article?id=10.1371/journal.pone.0122721, https://journals.plos.org/plosone/article?id=10.1371/journal.pone.0136787), but I was hoping to obtain permission to use/cite the official WWF ecoregion versions to ensure that all of my data and analyses are adequately cited and traceable.

I have attached the figure in which I hope to use the WWF ecoregions, which would appropriately cite the WWF in the figure caption and methods sections. I have also attached the example PLOS ONE permissions form showing what type of approval would be necessary. Please let me know if you are able to provide such approval, know of anyone who can, or require any additional information. It would be very much appreciated!

Thanks,
Tristan Frappier-Brinton
