# Supplementary material for: The burning island: Spatiotemporal patterns of fire occurrence in Madagascar
Source: PLoS One. 2022 Mar 31;17(3):e0263313. doi: 10.1371/journal.pone.0263313 (PMC8970516; doi:10.1371/journal.pone.0263313)
Supplement: Attachment — Submitted filename: comments_to_authors.pdf [file pone.0263313.s011.pdf]

## Overview

This study quantifies VIIRS hotspot detection, a proxy for fire use, on Madagascar, summarized by different ecoregions. It is well written and interesting There is no statistical modelling of drivers of fire use in different locations, which would provide further insight into fire use. However, the study provides an important summary/quantification of the issue, which sets the stage for greater future research. The authors are also clear about their method, their aim with the study and the discussion flows well from the results. The authors have summarized and presented the information well, and I believe this will be a valuable paper to researchers interested in human fire use, slash and burn agriculture and deforestation in the tropics.

I have several minor comments – mainly some clarification around certain text and some further explanation that is required to improve the manuscript.

## General comments

These are my main comments to the authors.

- In my opinion, you need to be clear how you are defining a "fire" in this paper. It looks like you are saying one hotspot is one fire, but one fire is usually defined (in fire behaviour/fire history studies) as the single continuous burnt area. That is, one wildfire can have multiple hotspots detected at once, e.g. in the picture below (VIIRS image overlayed with hotspots) I would say the green/yellow polygon (the eventual fire boundary) is one fire, and (at this particular time) there are multiple hotspots. So, I'd say you need to clearly define how you are using the word "fire", or change your use of fire to hotspot or another term in the text and in table 1 for example.

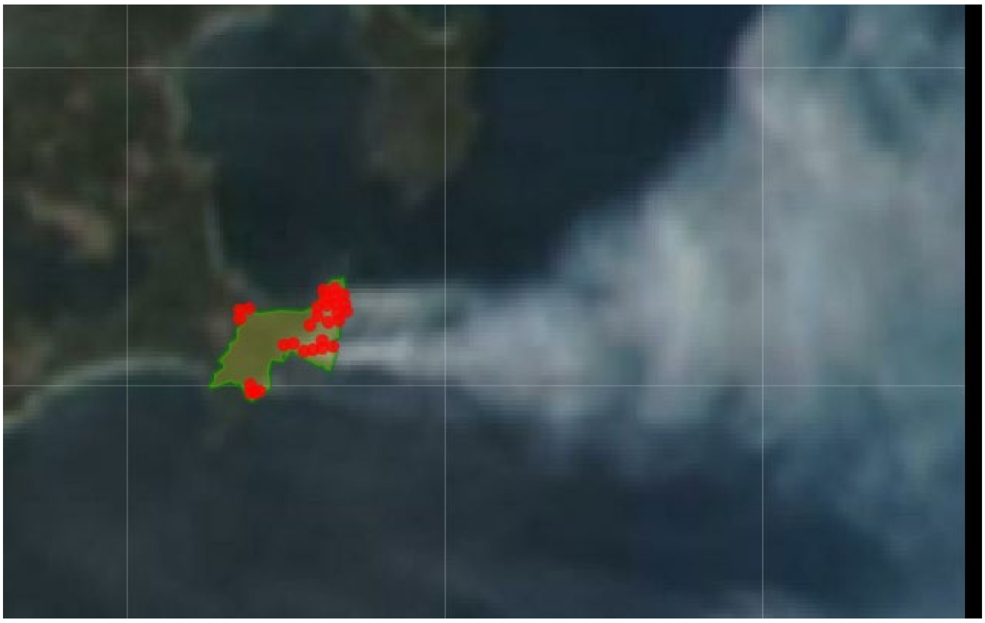

- Adding some explanation about how clouds may have affected the results is needed. Are there any areas/ecoregions of Madagascar that a consistently cloudier than other areas? If so, you could expect that VIIRS detection would be lower here because clouds may shielded fires from detection by VIIRS. This may be important for the results, e.g. if the lowlands are cloudier, than VIIRS may under-detect fires in this region. I think some acknowledgement of this issue in the methods or in section 4.5 is warranted.

## Specific comments

Below are my minor comments. Most are just around a bit of extra text required/some clarification that I believe would improve the manuscript.

### Line 42

It would be useful to add some specific examples of how or why fires have negative impacts on tropical forests (e.g. trees easily killed by fire because they are not adapted), rather than only the broad statement about "devastating effects" etc.

### Line 43

*"the most fire-threatened continent on Earth"*. I'd add this to the next paragraph, as the previous paragraph doesn't provide support for this statement.

### Line 100 and 102

What does "ca." represent?

### Line 102

The sentence starting *"In the southwest, the climate becomes"* has unclear wording. It sounds like the aphyllous shrubs continue south. I suggest rewording this sentence.

### Line 125 (Section 2.1)

Need to add a sentence or few words saying that there are two VIIRS instruments on separate satellites: Suomi NPP and NOAA-20. It is stated that you only used S-NPP, but it is worth saying there is another satellite now, but it is newer and had not collected data for your study period.

### Line 134

Similar to the previous point, better to clarify which satellite instrument. "VIIRS satellite" could refer to either, better to say VIIRS S-NPP or something similar.

### Line 144

Were there any industrial/urban areas false detection that needed to be excluded for your study area? Perhaps this was accounted for anyway when doing the intersection with the ecoregion polygons

### Line 236

Just a question, could this later peak be because mangroves take a long time to dry out, but when they do they are very flammable?

### Line 239

I believe the fig 3 caption should say "expressed in the number ***of*** fires per $km_2$ per month)

### Line 265

I found this chart a little confusing. This may be better as a bar chart i.e each buffer distance along the x axis and % along the y axis, and then a small bar for each ecoregion (repeated at each buffer distance). E.g. the "grouped bar chart" here: https://www.r-graph-gallery.com/48-grouped-barplot-with-ggplot2.html. Otherwise, add the points that make up each line so the buffer categories are clear.

### Line 267

Saying "that was within each ***buffer*** distance of the closest fire" would make this clearer.

## Line 271

Saying “effect” in the Table 1 caption implies a statistical model, where this just seems to be showing differences in summary statistics. I think you should say “Differences in distance to nearest fire by National park status” or something similar.

## Line 288

Reassuring in terms of what?

## Line 298

"*Analysis of the rate of change in fire frequency between 2012 and 2019 revealed that fire frequencies are increasing throughout the dry deciduous forests ecoregion*"
I suggest adding a table (or adding to an existing table) the mean change for each ecoregion type. I think this statement is just based of looking at the maps. It would be good to see some numbers.

## Line 360-366

As in the general comment, how does cloud influence this? Are the lowlands cloudier, so hotspots undetected there.

Also, you should not use the word “alarming” without explaining why. I would reword this final sentence (Line 366) to something like: If fires within forest are underestimated, the impact of fire on the forests is greater than can be determined/estimated by VIIRS detections..”, or something more similar.

## Line 369

Avoid “reassuring” without further explanation. Or just explain that it is a positive in terms of conservation outcomes

## Line 372

Is the 19.8% for non-national parks? Also probably better to say 2.8 times lower, rather than 2.8 fold decrease.

Also, could it be national parks are more commonly in less fire-prone areas, e.g. steep/hilly areas more inaccessible to farmers, therefore less fires ignited there due to the terrain rather than national park status. Add a note if you think that could be influencing the results.

## Line 389

What is guard density? Could you please clarify.
